# Dissection of Cell Death Induction by Wheat Stem Rust Resistance Protein Sr35 and Its Matching Effector AvrSr35

**DOI:** 10.1094/MPMI-08-19-0216-R

**Published:** 2019-12-16

**Authors:** Stephen Bolus, Eduard Akhunov, Gitta Coaker, Jorge Dubcovsky

**Affiliations:** 1 Department of Plant Sciences, University of California, Davis, CA 95616, U.S.A.; 2 Department of Plant Pathology, University of California, Davis; 3 Department of Plant Pathology, Kansas State University, Manhattan, KS 66506, U.S.A.; 4 Howard Hughes Medical Institute, Chevy Chase, MD 20815, U.S.A.

**Keywords:** AvrSr35, barley, cell death, disease resistance, effector, hypersensitive response, nucleotide-binding leucine-rich repeat receptor, *Puccinia graminis*, Sr35, stem rust, wheat

## Abstract

Nucleotide-binding leucine-rich repeat receptors (NLRs) are the most abundant type of immune receptors in plants and can trigger a rapid cell-death (hypersensitive) response upon sensing pathogens. We previously cloned the wheat NLR *Sr35,* which encodes a coiled-coil (CC) NLR that confers resistance to the virulent wheat stem rust race Ug99. Here, we investigated Sr35 signaling after *Agrobacterium-mediated* transient expression in *Nicotiana benthamiana*. Expression of Sr35 in *N. benthamiana* leaves triggered a mild cell-death response, which is enhanced in the autoactive mutant Sr35 D503V. The N-terminal tagging of Sr35 with green fluorescent protein (GFP) blocked the induction of cell death, whereas a C-terminal GFP tag did not. No domain truncations of Sr35 generated cell-death responses as strong as the wild type, but a truncation including the NB-ARC (nucleotide binding adaptor) shared by APAF-1, R proteins, and CED-4 domains in combination with the D503V autoactive mutation triggered cell death. In addition, coexpression of Sr35 with the matching pathogen effector protein AvrSr35 resulted in robust cell death and electrolyte leakage levels that were similar to autoactive Sr35 and significantly higher than Sr35 alone. Coexpression of Sr35-CC-NB-ARC and AvrSr35 did not induce cell death, confirming the importance of the leucine-rich repeat (LRR) domain for AvrSr35 recognition. These findings were confirmed through *Agrobacterium*-mediated transient expression in barley. Taken together, these results implicate the CC-NB-ARC domains of Sr35 in inducing cell death and the LRR domain in AvrSr35 recognition.

Plant diseases are a major source of crop losses worldwide (Cesari 2018). An environmentally sustainable approach to reduce these losses is to exploit plant natural resistance mechanisms (Nelson et al. 2018). Plants have evolved an elaborate innate immune system that relies on receptor-based direct or indirect detection of pathogen features and secreted virulence effector proteins (Thomma et al. 2011). Among the characterized plant resistance genes, the most abundant class are the intracellular nucleotide-binding leucine-rich repeat (NLR) receptors (Dangl et al. 2013). NLR proteins are activated by direct or indirect perception of pathogen effector proteins and are composed of variable N-terminal domains, a conserved nucleotide-binding (NB) domain, and a C-terminal leucine-rich-repeat (LRR) domain (Cesari 2018). A hallmark of NLR activation is a rapid programmed cell-death response termed the hypersensitive response (HR) (Chiang and Coaker 2015). However, other NLRs (e.g., wheat *Sr13* and *Sr21*) confer partial resistance by inducing the coordinated expression of multiple pathogenesis-related genes, which slow down infection without triggering an HR response (Chen et al. 2018; Zhang et al. 2017).

NLRs can be assigned to three main groups based on their N-terminal domains, namely, coiled-coil (CC), toll/interleukin-1 receptor-like (TIR), and a basal clade containing CC domains with similarity to RPW8 (CC_R_) NLRs (Shao et al. 2016). All plant lineages have CC and CC_R_ NLRs, whereas several lineages—including monocots—have lost the TIR NLRs (Shao et al. 2016). For CC NLRs, the CC domain is thought to play an important role in signaling. Indeed, the CC domains of multiple NLR proteins (MLA10, Sr33, Sr50, Rp1-D21, NRG1, ADR1, R3a, N’, I2, ZAR1, and Pvr4) are sufficient to induce cell death after transient expression in leaves of *Nicotiana benthamiana* (Bai et al. 2012; Baudin et al. 2017; Cesari et al. 2016; Collier et al. 2011; Hamel et al. 2016; Kim et al. 2018; Wang et al. 2015). However, there are other NLRs, including RPM1, Rx, Bs2, and RPS5, whose CC domains do not induce cell death in *N. benthamiana* (Ade et al. 2007; El Kasmi et al. 2017; Hamel et al. 2016). The CC domain of ZAR1 contributes to its oligomerization into a wheel-like pentamer (the resistosome) that is implicated in both the induction of cell death and disease resistance (Wang et al. 2019a). Some CC domains also play an important role in targeting NLRs to the cell membrane. For RPS5, alanine substitutions of predicted myristoylation and palmitoylation residues affected RPS5 plasma membrane localization, protein stability, and abolished cell death (Qi et al. 2012). Similarly, mutating two cysteines at an N-terminal predicted palmitoylation site to alanine blocked signaling and disrupted the membrane localization of the rice NLR protein Pit (Kawano et al. 2014).

The NB-ARC (nucleotide binding adaptor shared by APAF-1, R proteins, and CED-4) domain can be divided into three subunits, NB, ARC1, and ARC2 (Sukarta et al. 2016). These regions are thought to cooperate in nucleotide binding and coordinate intra- and intermolecular interactions that catalyze the switch between adenosine diphosphate and adenosine triphosphate binding (Collier and Moffett 2009). Upon effector perception, the NB domain of ZAR1 changes conformation and releases ADP to form an intermediate state (Wang et al. 2019b). Within the NB site, mutations in the highly conserved lysine of the P-loop motif (GxxxxGK[T/S]) greatly reduces the ability of NLR proteins to bind ATP and results in a signaling-inactive NLR protein (Tameling et al. 2002). Indeed, this lysine residue was shown to participate directly in ADP and ATP binding for ZAR1 (Wang et al. 2019a and b). In contrast, mutation of the conserved MHD motif to MHV (at the end of the ARC2 subunit) results in constitutively active NLR proteins (Bendahmane et al. 2002). Destabilization of ADP binding by alteration of the MHD motif may allow for domain reconfigurations leading to ATP-binding and activation (Tameling et al. 2006).

LRR domains are characterized by a repeating pattern of leucine or other hydrophobic amino acids (LxxLxLxxNxL). These repeats in plants fold into a parallel β-sheet and form an arc-shaped structure (Sukarta et al. 2016; Wang et al. 2019b). LRR domains have been implicated in both effector recognition and NLR auto-inhibition; they have hypervariable solvent-exposed amino acid residues, which may facilitate a diversity of intra- and intermolecular interactions (Sukarta et al. 2016). Indeed, the LRR domain of ZAR1 was found to play a key role in mediating effector recognition through protein-protein interactions, while also helping to inactivate ZAR1 in the absence of effector detection through intramolecular interactions (Wang et al. 2019b).

Wheat stem rust, caused by *Puccinia graminis* f. sp. *tritici* is a devastating disease. The wheat resistance gene *Sr35* encodes a CC NLR protein that confers near-immunity to Ug99 and related *P. graminis* f. sp. *tritici* races (Saintenac et al. 2013). The matching effector of Sr35, AvrSr35, is a protein of unknown function, and heterologous coexpression of Sr35 with AvrSr35 induced cell death in *N. benthamiana* (Salcedo et al. 2017). In this work, we characterized the induction of cell death by Sr35 in the presence and absence of AvrSr35, using *Agrobacterium*-mediated transient expression in *N. benthamiana* and *Hordeum vulgare* (barley). We show that overexpression of Sr35 triggers a weak cell-death response that is enhanced in the autoactive mutant in the MHD motif. No truncation variants of Sr35 were able to signal cell death at wild-type protein levels, but an MHD to MHV autoactive Sr35 CC-NB-ARC truncation was able to signal cell death. Sr35 coexpressed with AvrSr35 produced robust cell death, but coexpression of Sr35 CC-NB-ARC truncation with AvrSr35 did not. These results suggest a role of the CC-NB-ARC domains of Sr35 in the induction of cell death and the LRR domain in AvrSr35 recognition.

## RESULTS

### Overexpression of Sr35 triggers cell death in *Nicotiana benthamiana*.

**Fig. 1.**
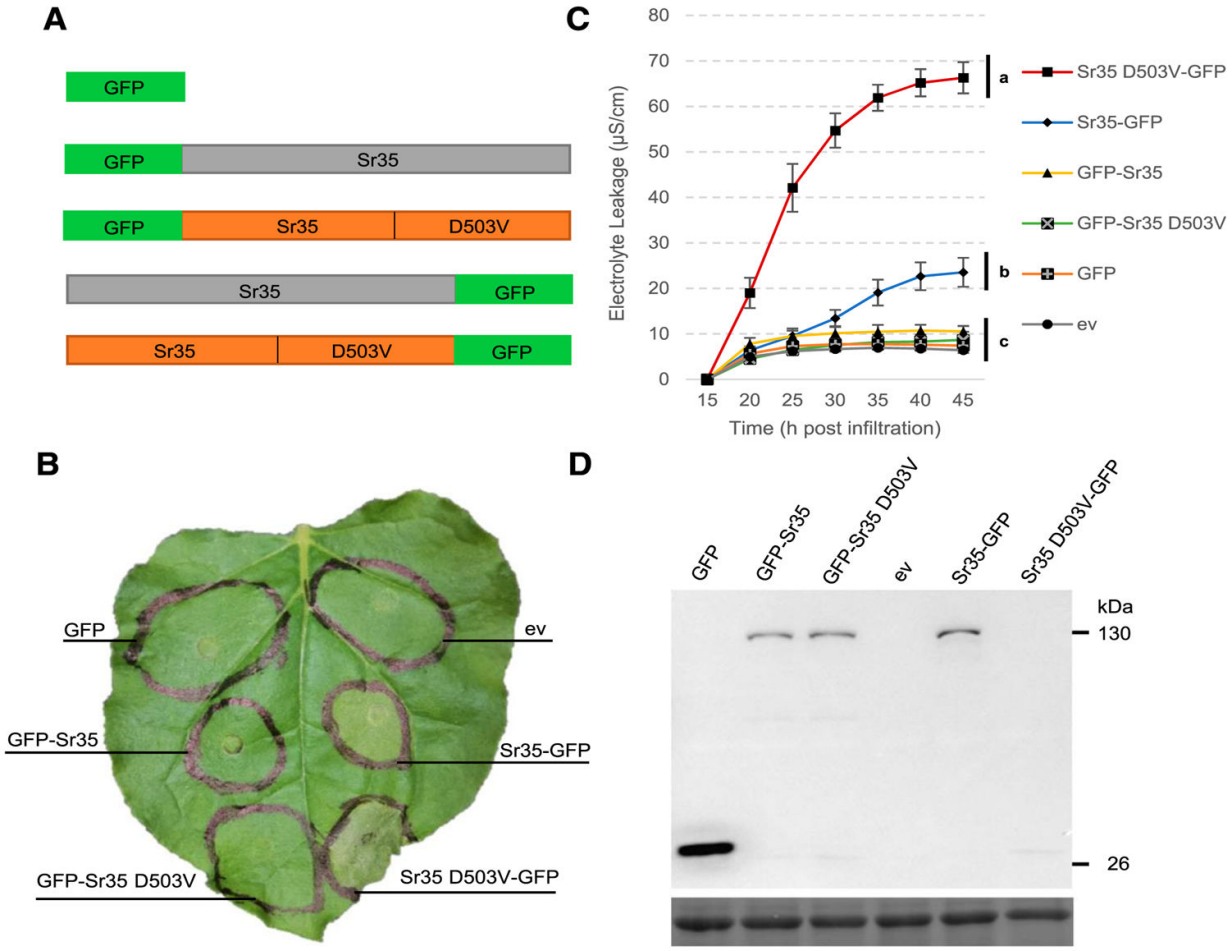
Sr35 induces cell death when overexpressed in *Nicotiana benthamiana*. **A,** Schematic diagram of green fluorescent protein (GFP) fused to the N (GFP-Sr35) and C (Sr35-GFP) terminus of Sr35 with and without the autoactive mutation (D503V). **B,** Macroscopic cell death observed 48 h postinfiltration (hpi) with *Agrobacterium tumefaciens*; ev = empty vector. **C,** Electrolyte leakage 15 to 45 hpi. Error bars represent standard error based on four biological replicates per construct. Different letters indicate significantly different groups of means based on Tukey’s honestly significant difference test (α= 0.01) performed at 45 hpi (statistical analyses in Supplementary Table S2). **D,** Western blots of protein extracts from *N. benthamiana* 24 hpi, analyzed using an anti-GFP-horseradish peroxidase antibody. No protein was observed here for Sr35 D503V-GFP, but it was detected after immunoprecipitation (Supplementary Fig. S2). The lower panel is the same blot stained with Ponceau S to reveal the Rubisco large subunit protein used as loading control.

In order to investigate Sr35 signaling, we sought to develop a system in which we could rapidly assess defense signaling from the Sr35 receptor. Transient overexpression of wheat Sr35 with a C-terminal green fluorescent protein (GFP) tag driven by the 35S promoter in *Agrobacterium*-infiltrated leaves of *N. benthamiana* resulted in a weak cell-death response that was sometimes difficult to detect macroscopically but was always confirmed in the more sensitive electrolyte leakage experiments described below. A similarly weak response was observed when the Sr35 protein was transiently overexpressed without the GFP tag (Supplementary Fig. S1). In contrast, robust cell death was observed after *N. benthamiana* infiltration with the Sr35 D503V-GFP autoactive mutant (Fig. 1B). Cell death was completely abolished when the GFP tag was placed at the N terminus, both in the wild type and autoactive versions of Sr35 (Fig. 1B). No macroscopic cell death was detected in the empty vector and GFP protein controls (Fig. 1B).

Macroscopic cell-death observations were confirmed and quantified by an electrolyte leakage experiment, which was repeated twice with similar results (Fig. 1C; Supplementary Table S2). A combined analysis of variance (ANOVA) using the two experiments as blocks and the two individual ANOVA by experiment showed statistically significant effects of the *Sr35* constructs on electrolyte leakage (*P* < 0.0001) (Supplementary Table S2). Mean comparisons using the Tukey test at 45 h postinfiltration (hpi) showed that the autoactive Sr35 D503V-GFP construct triggered electrolyte leakage levels more than twofold higher than the Sr35-GFP construct and sixfold higher than the other constructs and controls (*P* < 0.01). The electrolyte leakage levels of the Sr35-GFP construct were lower than those using the autoactive Sr35 D503V-GFP construct but were still twofold higher than the constructs with the N-terminal GFP tag and the negative controls (*P* < 0.01). Collectively, these experiments demonstrate that Sr35 is capable of triggering cell death in *N. benthamiana* and that this function is blocked by the addition of GFP to the N terminus.

In order to verify that all Sr35 proteins were expressed, we performed anti-GFP immunoblots. The results presented in Figure 1D confirmed that all proteins were expressed at the expected lengths except for Sr35 D503V-GFP, which was only detectable after immunoprecipitation (Supplementary Fig. S2). We also inferred that a functional Sr35 D503V-GFP protein was present based on the strong cell-death response generated by this construct (Fig. 1B). We speculate that the rapid degradation of most cell proteins triggered by the strong cell-death response may explain the low levels of protein detected in the Sr35 D503V-GFP autoactive sample.

### Truncation variants of Sr35 do not trigger cell death.

**Fig. 2.**
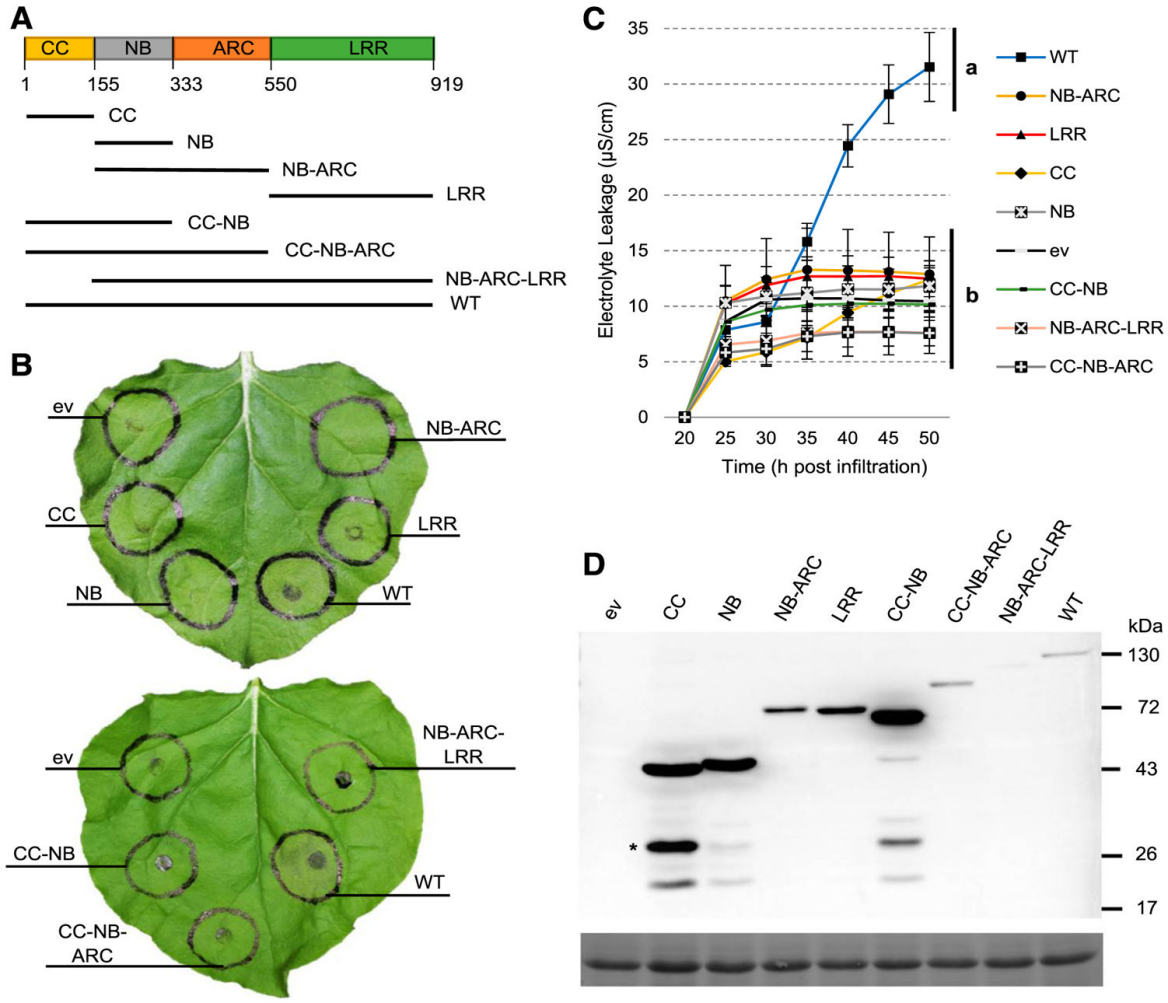
Truncation variants of Sr35 are not able to induce cell death. **A,** Model of Sr35 protein with amino acid numbers used to delineate the boundaries of domains and motifs listed below. CC = coiled-coil, NB = nucleotide binding, NB-ARC = nucleotide binding adaptor, and LRR = leucine-rich repeat. Sr35 wild-type (WT) protein and its truncated variants are shown below the model. **B,** Macroscopic cell death in *Nicotiana benthamiana* leaves 48 h postinfiltration (hpi) with Sr35 and its truncated variants. All proteins were C-terminally tagged with green fluorescent protein (GFP); ev refers to empty vector. **C,** Electrolyte leakage was monitored from 20 to 50 hpi. Error bars correspond to standard error based on four biological replicates per construct. Different letters represent significantly different groups of means, based on Tukey’s honestly significant difference test (α = 0.01) performed at the last timepoint (statistical analyses in Supplementary Table S3). **D,** GFP-horseradish peroxidase Western blot showing proteins expressed at expected sizes for all constructs. GFP cleavage (*) was observed in CC, NB, and CC-NB constructs. The lower panel is the same blot stained with Ponceau S to reveal bands corresponding to the Rubisco large subunit protein as loading control.

To determine the region of Sr35 required for inducing cell death in *N. benthamiana*, we generated constructs over-expressing Sr35 protein domains individually and in different combinations with C-terminal GFP tags (Fig. 2A). Overexpression of the CC domain of Sr35 in *N. benthamiana* with (Fig. 2B) or without (Supplementary Fig. S1) the C-terminal GFP tag showed no macroscopic cell death. Likewise, none of the other Sr35 domains or domain combinations generated macroscopic cell death (Fig. 2B). These results were confirmed by the electrolyte leakage induced by overexpression of these constructs (Fig. 2C; Supplementary Table S3). Highly significant differences (*P* < 0.0001) were detected in the ANOVA tests among the different constructs, both in the individual experiments and in the combined analysis using experiments as blocks (Supplementary Table S3). Comparisons among electrolyte leakage means using the Tukey tests revealed highly significant differences between the wild-type Sr35 and all the Sr35 truncations (*P* < 0.01) in both the individual experiments and the combined ANOVA (Supplementary Table S3).

In order to verify that Sr35 and the truncation variants were expressed in our constructs, we performed anti-GFP immunoblots. These experiments revealed proteins at the expected sizes; however, cleaved GFP was also observed in Sr35 CC, NB, and CC-NB constructs, possibly due to the relatively high expression levels of these constructs (Fig. 2D). Taken together, these results indicate that truncation of any of the domains from wild-type Sr35 protein abolishes the cell-death response observed with the full-length protein.

### Sr35 CC-NB-ARC with the D503V autoactive mutation is capable of signaling.

**Fig. 3.**
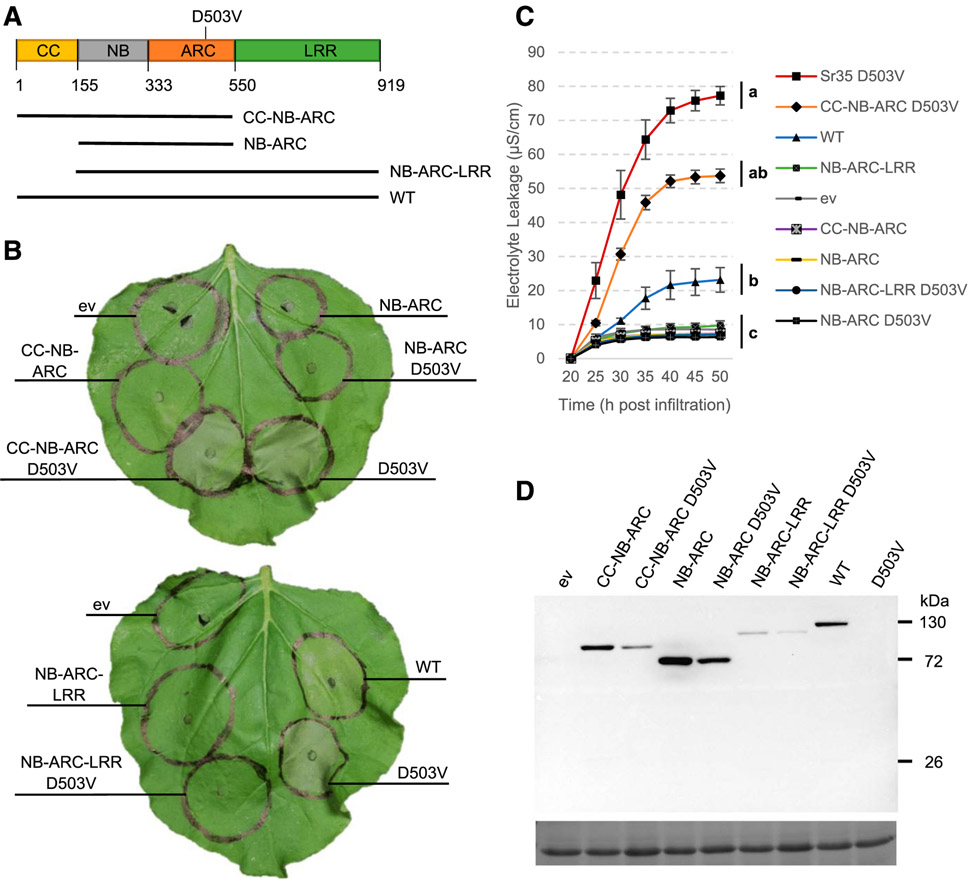
Sr35 coiled-coil nucleotide binding adaptor (CC-NB-ARC) D503V induces strong cell death. **A,** Model of Sr35 protein with the amino acid numbers used to delineate the boundaries of domains and motifs listed below. NB = nucleotide binding and LRR = leucine-rich repeat. The D503Vautoactive mutation is marked above the model. Truncation variants used in this experiment are shown below. **B,** Macroscopic cell death in *Nicotiana benthamiana* leaves 48 h postinfiltration (hpi) with *Agrobacterium tumefaciens*; ev refers to empty vector construct. **C,** Electrolyte leakage for all constructs was monitored 20 to 50 hpi. Error bars represent standard error based on four biological replicates per construct. Different letters represent significantly different groups of means, based on Tukey’s honestly significant difference test (α = 0.01) performed at the last timepoint (statistical analyses in Supplementary Table S4). **D,** Green fluorescent protein (GFP) horseradish peroxidase Western blot showing that all constructs except Sr35 D503V-GFP expressed proteins of the expected sizes. Sr35 D503V-GFP was detected by immunoprecipitation (Supplementary Fig. S2). Relatively weak protein expression was noted for all constructs with the D503V autoactive mutation. The lower panel is the same blot stained with Ponceau S to reveal bands corresponding to the Rubisco large subunit protein as loading control.

To test if the presence of the D503V autoactive mutation could enhance the ability of the truncation variants to induce cell death, we introduced the D503V mutation into the Sr35 NB-ARC, CC-NB-ARC, and NB-ARC-LRR constructs (Fig. 3A). Overexpression of these constructs in *N. benthamiana* revealed that the CC-NB-ARC construct with the D503V autoactive mutation induced macroscopic cell death by 48 hpi (Fig. 3B).

Analysis of the electrolyte leakage levels in *N. benthamiana* leaves transiently over-expressing these constructs at 50 hpi revealed significant differences in the ANOVA of two independent experiments and in the combined analysis using experiments as blocks (*P* < 0.0001, Supplementary Table S4). Comparisons among means using a Tukey test (*P* < 0.01) showed that the leakage levels induced by the full-length Sr35 D503V protein, the Sr35 CC-NB-ARC D503V truncation, and the Sr35 wild type were significantly higher than the levels induced by all the other constructs (Fig. 3C; Supplementary Table S4). Among these three constructs, the full-length Sr35 D503V showed the highest electrolyte leakage, but the differences with Sr35 CC-NB-ARC D503V were not significant in any of the analyses. While wild-type Sr35 showed significantly lower leakage values than Sr35 D503V and CC-NB-ARC D503V in the combined ANOVA (*P* < 0.01), the differences in the individual experiments were significant only between Sr35 wild type and Sr35 D503V (Supplementary Table S4).

Experiments with anti-GFP immunoblots confirmed the presence of proteins of the expected sizes for all constructs (Fig. 3D). There was a clear trend for all three truncated constructs carrying the D503V mutation to accumulate lower protein levels than the corresponding construct without the autoactivating mutation (Fig. 3D). Consistent with the previous result, the complete Sr35 D503V-GFP was only detectable after immunoprecipitation (Supplementary Fig. S2). Altogether, our results indicate that the Sr35 CC-NB-ARC, when coupled with the autoactivating D503V MHD mutation, is sufficient for initiating cell death.

### Mutations in predicted palmitoylation residues do not affect signaling.

In a previous study, we showed that Sr35 colocalized with a marker for the endoplasmic reticulum (Salcedo et al. 2017). Since association to cell membranes can be facilitated by the presence of palmitoylation sites, we searched for palmitoylation motifs in Sr35, using the CSS-Palm4.0 prediction website. Two potential palmitoylation sites were detected at cysteine residues 319 (score 2,485, threshold 2,412) and 649 (score 7,315, threshold 3,717) in the NB and LRR domains, respectively (Supplementary Fig. S3). We introduced mutations at these positions that resulted in cysteine to alanine substitutions in the wild-type and D503V autoactive Sr35 proteins. The constructs with the mutations in the predicted palmitoylation sites induced cell-death responses similar to their corresponding constructs without the mutations (Supplementary Fig. S3). The empty vector and the Sr35 K206R P-loop mutant, included as negative controls, showed no induction of cell death (Supplementary Fig. S3).

Individual ANOVA analyses and a combined ANOVA, in which two individual experiments were analyzed as blocks, revealed significant differences in the *Sr35* constructs tested for electrolyte leakage (*P* < 0.0001) (Supplementary Fig. S3; Supplementary Table S5). The two constructs with the D503V autoactivating mutations showed significantly higher electrolyte leakage levels (Tukey test, *P* < 0.01) (Supplementary Table S5) than all other constructs in both single experiment and combined ANOVA analyses. The wild-type constructs (with and without the mutations at the putative palmitoylation sites) showed significantly higher leakage levels than the empty vector and K206R P-loop mutant in both single experiment and combined ANOVA analyses (more than threefold higher, Tukey test, *P* < 0.01) (Supplementary Table S5).

Anti-GFP immunoblots confirmed the expression of proteins of the expected sizes (Supplementary Fig. S3). The complete constructs with the autoactive mutation (D503V and C319A/C649A/D503V) were not detected in the anti-GFP immunoblots but were detected after immunoprecipitation (Supplementary Fig. S2).

### Sr35 induces cell death in barley.

**Fig. 4.**
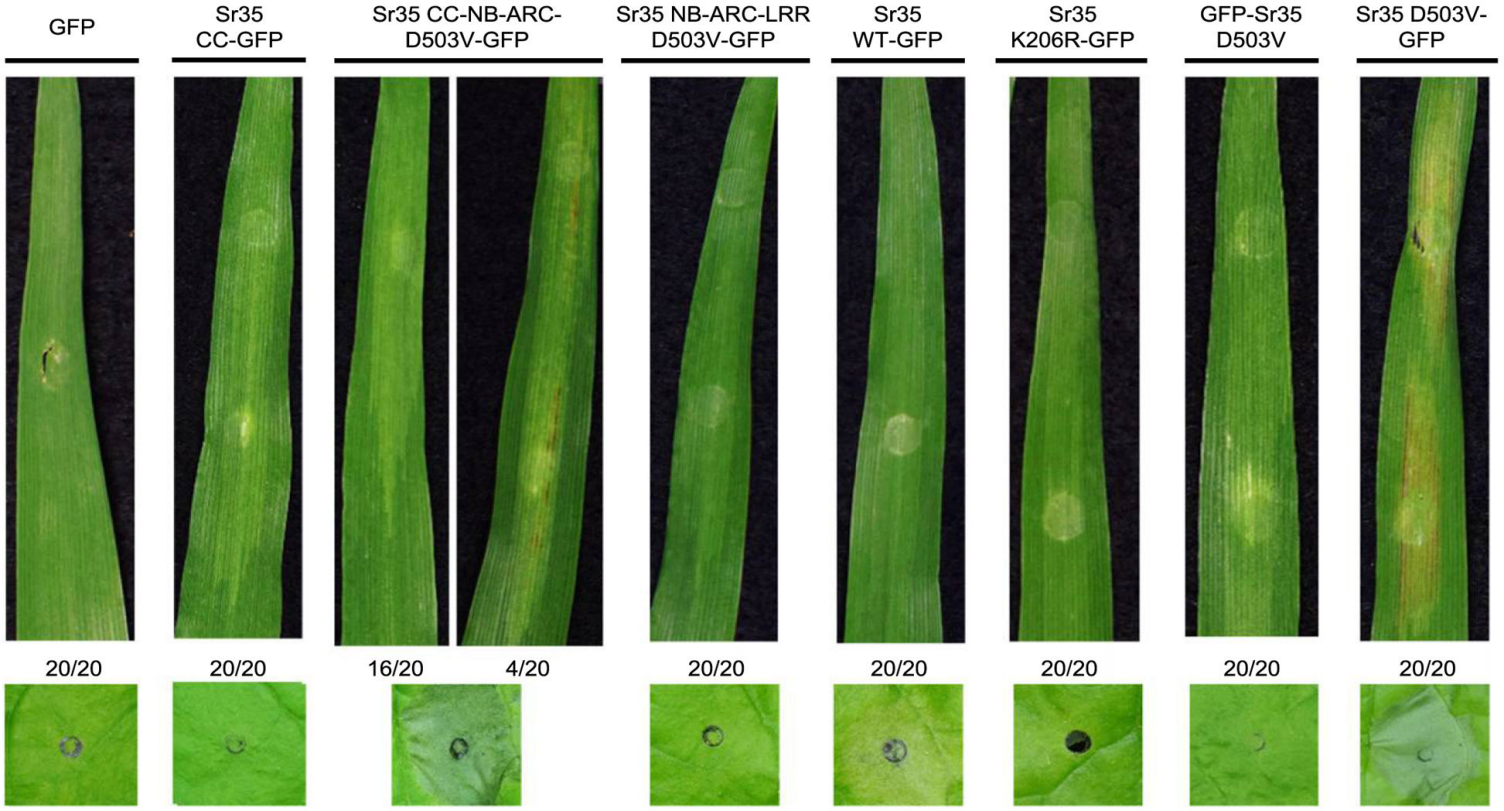
Transient expression of *Sr35* constructs in barley. Top panels, macroscopic cell death in Manchuria barley leaves 7 days postinfiltration with *Agrobacterium tumefaciens* carrying *Sr35* constructs. Ratios represent the number of the 20 leaves transformed with each construct that showed the same result as the presented images. Lower panels, macroscopic cell death in *Nicotiana benthamiana* leaves 48 h postinfiltration with the same *Agrobacterium* culture. Leaf images were cropped to save space. The entire experiment was repeated twice with similar results.

It has been shown recently that Sr35 can confer resistance to *P. graminis* f. sp. *tritici* in barley (Hatta et al. 2018). Using a method for *Agrobacterium* infiltration described previously (Lu et al. 2016), we tested our constructs for signs of macroscopic cell death in barley. Similar to observations in *N. benthamiana*, Sr35 D503V-GFP induced a consistent cell-death response in barley but GFP-Sr35 D503V did not (Fig. 4). However, macroscopic cell death was not observed in barley for the wild-type Sr35-GFP construct as it was in *N. benthamiana* infiltrated with the same bacterial culture (Fig. 4). Sr35 CC-NB-ARC D503V-GFP did not induce as strong a cell-death response in barley as in *N. benthamiana*, but several of the infiltrated leaves (four of 20) showed signs of macroscopic cell death (Fig. 4), and this result was reproducible between experiments. This could be due to differences in protein expression levels between the two systems, because the 35S promoter used in these experiments is more effective in dicot than in monocot species (Christensen et al. 1992). Similar to the results observed in *N. benthamiana*, GFP, Sr35 CC-GFP, Sr35 K206R-GFP, and Sr35 NB-ARC-LRR D503V-GFP did not trigger cell death in barley (Fig. 4). These results suggest that Sr35 exhibits similar signaling mechanisms in both monocots and dicots.

### AvrSr35-induced cell death is restricted to C-terminally tagged, full-length Sr35 protein.

**Fig. 5.**
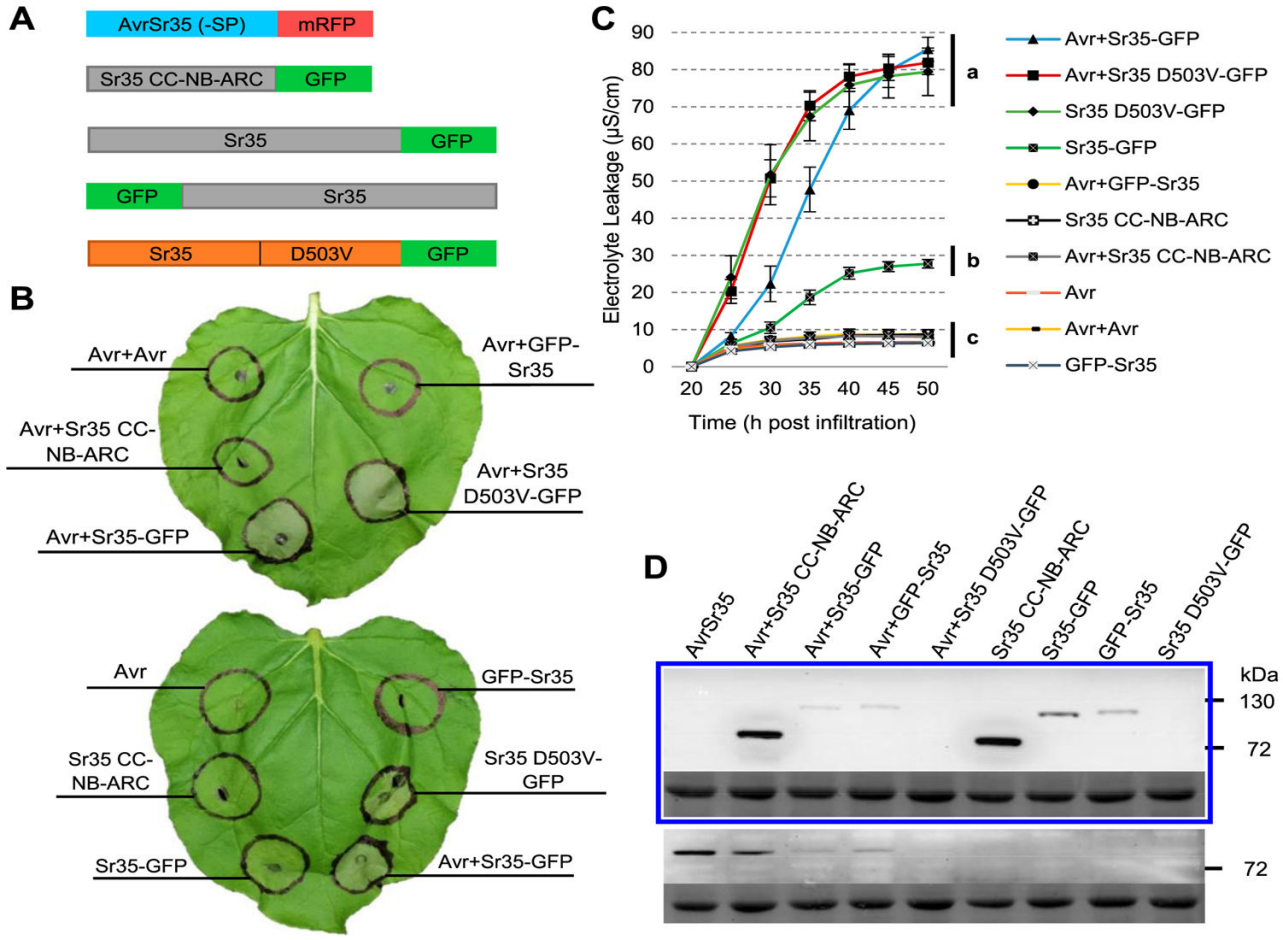
AvrSr35 and Sr35 with a C-terminal green fluorescent protein (GFP) tag trigger a strong cell-death response. **A,** Schematic diagram of *Sr35* and *AvrSr35* constructs. **B,** Macroscopic cell death in *Nicotiana benthamiana* leaves 48 h postinfiltration (hpi) with *Agrobacterium tumefaciens* (+ indicates coinfiltration). Avr refers to the AvrSr35–SP+mRFP construct (no signal peptide and monomeric red fluorescent protein [mRFP] C-terminal tag). Avr+Avr coinfiltration control corresponds to AvrSr35 infiltrated at double the optical density. **C,** Electrolyte leakage for all constructs was monitored from 20 to 50 hpi. Error bars represent standard error based on four biological replicates per construct. Different letters represent significantly different groups of means, based on Tukey’s honestly significant difference test (α = 0.01) performed at the last timepoint (statistical analyses in Supplementary Table S6). **D,** GFP-HRP (horseradish peroxidase) Western blot (upper panel) showing that all constructs except Sr35 D503V-GFP expressed proteins of the expected sizes. Sr35 D503V-GFP was detected by immunoprecipitation (Supplementary Fig. S2). The blot shown below is the same blot stained with Ponceau S to reveal bands corresponding to the Rubisco large subunit protein as a loading control. DsRed and mouse-HRP Western blot (lower panel) revealed AvrSr35 in all coinfiltrated samples except Avr+Sr35 D503V-GFP, likely due to rapid cell death. The blot shown below is the same blot stained with Ponceau S to reveal bands corresponding to the Rubisco large subunit protein as loading control.

Sr35 and AvrSr35 were previously shown to trigger cell death when coexpressed in *N. benthamiana* (Salcedo et al. 2017). To test if the CC-NB-ARC truncation is capable of inducing cell death in the presence of AvrSr35, we coexpressed Sr35 CC-NB-ARC-GFP, Sr35-GFP, and GFP-Sr35 with an AvrSr35 protein that lacked the signal peptide and included a C-terminal monomeric red fluorescent protein (mRFP) tag (AvrSr35–SP+mRFP) (Fig. 5A). We included Sr35 D503V-GFP as a positive control. Full-length Sr35-GFP coexpressed with AvrSr35–SP+mRFP induced macroscopic cell-death reactions similar to those observed in autoactive Sr35 D503V-GFP in *N. benthamiana* (Fig. 5B). Sr35-GFP, when expressed alone, induced a mild cell-death response, and no macroscopic effects were detected for the other constructs.

In order to quantify and compare cell-death responses, statistical analyses were performed on electrolyte leakage data collected at 50 hpi (Fig. 5C). Significant effects were detected in the combined ANOVA using the two individual experiments as blocks as well as in the two individual ANOVA (*P* < 0.0001) (Supplementary Table S6). Mean comparisons using Tukey tests (*P* < 0.01) showed that coexpression of Sr35-GFP and AvrSr35–SP+mRFP induced electrolyte leakage levels that were not significantly different from autoactive Sr35 D503V-GFP alone or autoactive Sr35 D503V-GFP coexpressed with AvrSr35–SP+mRFP (Fig. 5C). These constructs induced significantly higher electrolyte leakage levels than all other constructs in both individual and combined analyses (*P* < 0.01). Sr35-GFP alone induced lower electrolyte leakage levels than the autoactive constructs and Sr35-GFP coinfiltrated with AvrSr35–SP+mRFP (*P* < 0.01) but was significantly higher (*P* < 0.01) than all other constructs in both the individual and combined analyses (*P* < 0.01) (Fig. 5C; Supplementary Table S6).

Sr35 proteins were expressed at the expected sizes as revealed in an anti-GFP immunoblot (Fig. 5D). The same protein samples were tested for AvrSr35–SP+mRFP, using anti-DsRed immunoblot. AvrSr35–SP+mRFP was expressed in coinfiltration samples as expected (Fig. 5D). However, AvrSr35–SP+mRFP was hardly detected when coinfiltrated with Sr35 D503V-GFP, likely due to rapid cell death. As before, Sr35 D503V-GFP was only detected after immunoprecipitation (Supplementary Fig. S2).

**Fig. 6.**
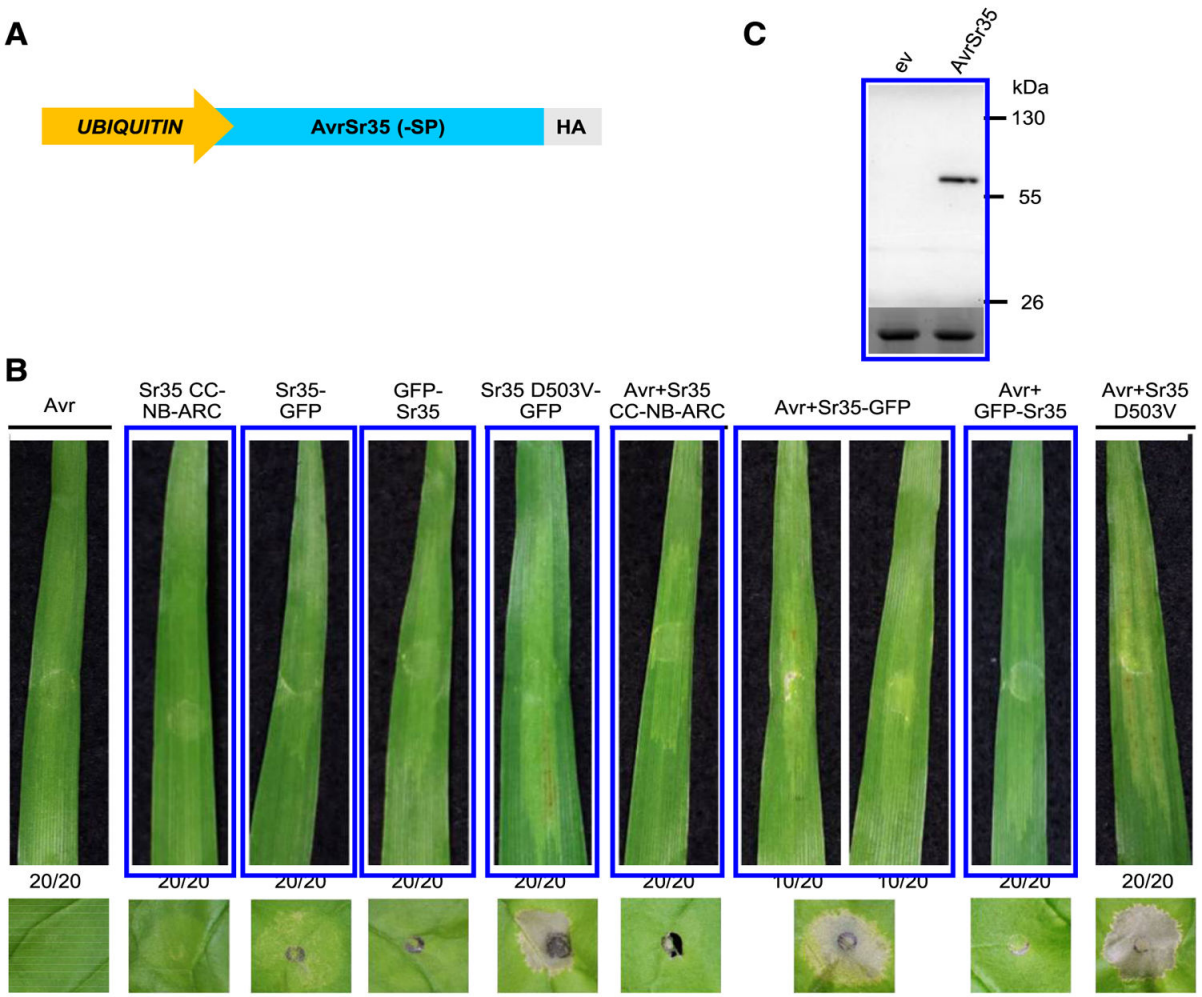
AvrSr35 recognition by Sr35 in barley. **A,** Model of AvrSr35–SP+HA (no signal peptide and C-terminal hemagglutinin [HA] tag) construct. **B,** Transient expression of Sr35 and AvrSr35–SP+HA constructs in barley. Top panel: macroscopic cell death in Manchuria barley leaves 7 days postinfiltration with *Agrobacterium tumefaciens* carrying Sr35 and AvrSr35–SP+HA constructs. Ratios represent the number of the 20 leaves transformed with each construct that showed the same result as the presented images. Lower panel: macroscopic cell death in *Nicotiana benthamiana* leaves 72 h postinfiltration with the same *Agrobacterium* culture. Leaf images were cropped to save space. The experiment was repeated twice with similar results. **C,** HA-horseradish peroxidase Western Blot (upper panel) showing AvrSr35–SP+HA expressed at the expected size in *N. benthamiana*. ev = empty vector. The blot was also stained with Ponceau S (lower panel) to reveal bands corresponding to the Rubisco large subunit protein as a loading control.

To test the effect of coexpression of AvrSr35 and Sr35 in barley using *Agrobacterium*-mediated transient expression, we cloned AvrSr35–SP downstream of a maize *UBIQUITIN* promoter and upstream of a 3×HA (hemagglutinin) tag (Fig. 6A). Coexpression of Sr35-GFP with AvrSr35–SP+3×HA in barley resulted in macroscopic cell death in 10 of the 20 leaves tested from different plants (Fig. 6B), whereas the autoactive construct Sr35 D503V-GFP with and without AvrSr35–SP+3×HA induced cell death in all 20 leaves tested. Macroscopic cell death was not observed for GFP-Sr35 and Sr35 CC-NB-ARC with and without AvrSr35–SP+3×HA (Fig. 6B). As controls, AvrSr35–SP+mRFP and Sr35-GFP overexpressed alone did not trigger cell death (Fig. 6B). For comparison, leaves of *N. benthamiana* were infiltrated with the same bacterial cultures as barley. Macroscopic cell death was consistent between *N. benthamiana* and barley except for Sr35-GFP, for which a weak cell death was observed only in *N. benthamiana* (Fig. 6B). AvrSr35–SP+3×HA expression was tested in *N. benthamiana*, using an anti-HA immunoblot. The AvrSr35 protein was expressed at the expected size (Fig. 6C).

Taken together, these results confirmed that AvrSr35 is able to induce activation of Sr35 in *N. benthamiana*, and we extended this observation to barley. In addition, we show that Sr35 recognition of AvrSr35 and the subsequent induction of cell death is dependent on the presence of the LRR domain and is blocked by a GFP tag on the N terminus of the Sr35 protein.

## DISCUSSION

The results presented in this study provide evidence that Sr35 is capable of inducing cell death in the presence of the matching AvrSr35 effector in both *N. benthamiana* and barley. Furthermore, we identified truncation variants and point mutations in the Sr35 protein that enhanced or reduced its ability to trigger cell death.

### Point mutations and fusion tags affecting the ability of Sr35 to signal cell death.

The difference between the mild cell-death response induced by the wild-type Sr35 construct (Fig. 1B) and the strong cell-death response observed with the coinfiltration of Sr35 and AvrSr35 (Fig. 5B) suggests that the wild-type Sr35 protein is present mainly in an inactive state and can be activated by the AvrSr35 effector in a heterologous system. The mild electrolyte leakage induced by the wild-type construct may be the result of the strong overexpression of Sr35 driven by the 35S promoter in *N. benthamiana*. This overexpression could result in the accumulation and autoactivation of Sr35 proteins due to improper intra- or intermolecular interactions. The 35S promoter used to overexpress Sr35 protein in *N. benthamiana* is less effective in monocots (Christensen et al. 1992), which may explain the absence of a macroscopic cell-death response when the same construct was used to express Sr35 in barley leaves (Fig. 4).

The hypothesis that the Sr35 protein in *N. benthamiana* and barley is mainly present in an inactive state is supported by the stronger HR observed in both species when the autoactivating mutation D503V is incorporated into the Sr35 full-length constructs (Figs. 1 and 4). The MHD to MHV mutation results in constitutively active NLR proteins from many plant species (Bai et al. 2012; Bendahmane et al. 2002; Gao et al. 2011; Kawano et al. 2014; Tameling et al. 2006). In contrast, the Sr35 P-loop mutant K206R reduced cell death triggered by the overexpression of Sr35 in *N. benthamiana* (Supplementary Fig. S3). This is consistent with other findings in which a mutation of the conserved lysine in the P-loop motif to arginine leads to NLR inactivation (Bai et al. 2012; Tameling et al. 2006). Mutation of two predicted palmitoylation motifs in Sr35 protein did not affect cell-death responses of WT or autoactive proteins (Supplementary Fig. S3), suggesting that either they were not real palmitoylation sites or that their presence is not essential for inducing cell death. This was contrary to what was observed in Pit, a rice NLR protein conferring resistance to the blast fungus, in which mutation of the two predicted palmitoylation motifs in the CC domain led to inactivation of the NLR protein (Kawano et al. 2014).

Both Sr35 and Sr35 D503V autoactive constructs lost their ability to trigger cell death in *N. benthamiana* (Fig. 1) and barley (Fig. 4) when a GFP tag was added to the N-terminal domain but not when it was added to the C-terminal domain. A similar loss-of-function when a tag was fused to the N terminus but not when it was fused to the C terminus was reported for Pit (Kawano et al. 2014), ZAR1 (Wang et al. 2019a), and both MLA1 and MLA6 (Bieri et al. 2004). In contrast, RPM1 was still able to trigger cell death with the addition of an N-terminal CBL membrane–targeting tag (Gao et al. 2011). The addition of an N-terminal tag may affect the ability of these NLRs to function properly by interfering with their ability to associate with membranes. Indeed, the addition of an N-terminal tag to Pit changed its localization from membrane-associated to cytosolic (Kawano et al. 2014) and changes in the CC domain leading to membrane localization and subsequent NLR activation was important for oligomerization and cell death in the CC NLR ZAR1 (Wang et al. 2019a).

### Ability of different Sr35 domains and domain combinations to signal cell death.

The ability of an N-terminal tag to suppress Sr35 induction of cell death indicates that the N-terminal CC domain may play an important role in this process. Our experiments using different Sr35 truncated proteins were consistent with this hypothesis. The truncation of the CC domain from the complete Sr35 D503V autoactive mutant eliminated the strong HR and high electrolyte leakage levels induced by this construct (Fig. 3B). Similarly, the CC-NB-ARC D503V construct lost its ability to induce cell death when the CC domain was not included in the construct (Fig. 3B). These results, together with the inability of the CC domain alone to induce cell death in the leaves of *N. benthamiana* (Fig. 2B), suggest that the CC domain of Sr35 plays a critical role in the induction of cell death but is not sufficient to induce this response.

No Sr35 truncation was able to match the strength of cell death triggered by the full-length Sr35 in *N. benthamiana* (Fig. 2). Several domains or domain combinations that were sufficient to induce cell death in other CC-NLR proteins were not effective for Sr35. For example, the CC-NB construct was not able to induce cell death in Sr35 but was sufficient to induce HR for Rx, MLA10, Sr33, and Sr50 (Bai et al. 2012; Cesari et al. 2016; Rairdan et al. 2008). Similarly, the CC-NB-ARC domain combination was unable to trigger cell death in Sr35 but was effective for RPS5, MLA10, and Pvr4 (Ade et al. 2007; Bai et al. 2012; Kim et al. 2018). However, we observed a strong cell-death signal for the Sr35 CC-NB-ARC D503V autoactive construct, whereas the same domain and mutant combination was not effective for RPM1 (El Kasmi et al. 2017). This result suggests that the CC-NB-ARC domains are sufficient to generate active folding of the Sr35 protein, but this is not a universal phenomenon for CC-containing NLRs.

### Sr35 requires the LRR domain to signal cell death in response to AvrSr35.

Although the autoactive mutant Sr35 CC-NB-ARC D503V truncation was able to induce cell death (Fig. 3B), coexpression of Sr35 CC-NB-ARC with AvrSr35 did not result in cell death in either *N. benthamiana* (Fig. 5) or barley (Fig. 6). This finding implicates the LRR domain in AvrSr35 recognition but decouples recognition from Sr35 signaling activity. An N-terminal tag on the Sr35 protein was able to block the induction of cell death in the presence of AvrSr35 (Fig. 5B). This was consistent with the previous finding that Sr35 D503V with an N-terminal GFP tag is not capable of triggering cell death (Fig. 1B). Therefore, our current model is that Sr35 is activated (directly or indirectly) by AvrSr35 through its LRR domain and that this recognition leads to changes in the CC-NB-ARC domains that facilitate signaling. This model is consistent with the observation that the in planta interaction between AvrSr35 and Sr35 is negatively affected by three amino acid substitutions at positions 854, 856, and 858 in the LRR domain of Sr35 and the susceptibility of this mutant to the Ug99 race of stem rust pathogen (Saintenac et al. 2013; Salcedo et al. 2017). The importance of the distal region of the LRR in pathogen recognition is also supported by an induced mutation (W856*) that eliminates the last 63 amino acids of the Sr35 LRR and results in susceptible plants (Saintenac et al. 2013).

## MATERIALS AND METHODS

### Plasmid construction.

The *Triticum monococcum* Sr35 genomic sequence without a stop codon was PCR-amplified from a clone previously used in transgenic complementation experiments (Saintenac et al. 2013), using Phusion polymerase (New England BioLabs) and primer pair Sr35gateway_F/R (Supplementary Table S1). Sr35 CC, CC-NB, CC-NB-ARC, NB, NB-ARC, NB-ARC-LRR, and LRR fragments were PCR-amplified from the same genomic clone using primer pairs Sr35gateway_F/Sr35CCgateway_R, Sr35gateway_F/Sr35NBgateway_R, Sr35gateway_F/Sr35ARCgateway_R, Sr35NBgateway_F/Sr35NBgateway_R, Sr35NBgateway_F/Sr35ARCgateway_R, Sr35NBgateway_F/Sr35gateway_R, and Sr35LRRgateway_F/Sr35gateway_R, respectively (Supplementary Table S1). PCR products were inserted into Gateway pDONR/Zeo vector using Gateway BP Clonase II enzyme mix (Thermo Fisher).

Single and multiple mutation constructs were made using PCR site-directed mutagenesis and, if necessary, multiple rounds of PCR site-directed mutagenesis on pDONR constructs. The CSS-Palm 4.0 server (Ren et al. 2008) was used to predict the putative palmitoylation sites, and Sr35 PCR primer pairs Sr35C319A_F/ R and Sr35C649A_F/ R (Supplementary Table S1) were used to induce mutations to replace the cysteines in the predicted palmitoylation sites with alanines. The D503V autoactive mutant and the K206R P-loop mutant constructs were generated using PCR primer sets Sr35D503V_F/R and Sr35K206R_F/R, respectively (Supplementary Table S1). C-terminal stop codons in Sr35 and Sr35 CC constructs were generated with primer pairs Sr35gateway_F/Sr35_TGA_gateway_R and Sr35gateway_F/Sr35CC_TGA_gateway_R (Supplementary Table S1). For all constructs, mutations were verified by Sanger sequencing. Sr35 and Sr35 D503V were transferred to the pGWB5 (GFP-C-terminal) and pGWB6 (GFP-N-terminal) destination binary vectors (Nakagawa et al. 2007) using Gateway LR Clonase II enzyme mix (Thermo Fisher). All other constructs were transferred to the pGWB5 vector only.

The pSITE4NA AvrSr35(−SP)+mRFP construct was provided by E. Akhunov (Salcedo et al. 2017) and was verified by Sanger sequencing. The AvrSr35 without signal peptide (AvrSr35–SP) in pLC41HA construct was made by first amplifying AvrSr35(−SP) with primer pair AvrSr35gateway_F/R (Supplementary Table S1). The resulting PCR product was inserted into Gateway pDONR/Zeo vector using Gateway BP Clonase II enzyme mix (Thermo Fisher). The construct sequence was verified by Sanger sequencing and was transferred downstream of the maize UBIQUITIN promoter and upstream of a 3×HA tag in a modified, Gateway-compatible pLC41 (Japan Tobacco) destination binary vector using LR Clonase II enzyme mix (Thermo Fisher).

### Transient expression in *Nicotiana benthamiana*.

*Nicotiana benthamiana* plants were grown for 3 weeks in growth chambers at 25°C with 85% humidity and 16 h of light at 100 μmol s^−1^ m^−2^. *Agrobacterium tumefaciens* EHA105 bacteria were transformed with pGWB5 and pGWB6 *Sr35* constructs. Transformed *A. tumefaciens* were cultured overnight at 28°C in Luria Bertani (LB) broth containing 50 μg of kanamycin or spectinomycin and 100 μg of rifampicin per milliliter. Bacteria were pelleted by centrifugation and were resuspended in infiltration media (1.15× Murashige and Skoog basal salts, 58 mM sucrose, 10 mM MES (pH 5.6), and 200 μmol acetosyringone). *A. tumefaciens* culture density was then adjusted to an optical density at 600 nm (OD_600_) of 0.5 and incubated with shaking at room temperature for at least 1 h prior to infiltration in *N. benthamiana* leaves using a 1-ml tuberculin syringe. For Sr35-AvrSr35 coinfiltration experiments, *A. tumefaciens* cultures were adjusted to OD_600_ 1.0 for each construct, then, mixed 1:1 with another construct or infiltration media (OD_600_ 0.5 for each construct after mixing). After *Agrobacterium* infiltration, *N. benthamiana* plants were placed at room temperature under constant 30 μmol s^−1^ m^−2^ light. Leaf tissue for electrolyte leakage was harvested starting at 15 or 20 hpi. Leaf tissue for protein extraction was sampled 24 hpi, and leaves for macroscopic cell death were sampled and imaged at 48 hpi.

### Electrolyte leakage assay.

To quantify cell death, leaf discs (0.79 cm^2^) of *Agrobacterium*-infiltrated *N. benthamiana* leaves were made using a cork borer and were placed as pairs (two subsamples from the same leaf) in 12-well tissue culture plates (VWR) with 5 ml of distilled water for 30 min. Four biological replicates (an individual, *Agrobacterium*-infiltrated leaf on an individual *N. benthamiana* plant) were made for each *Agrobacterium* culture. Water was replaced with 5 ml of new distilled water, and electrolyte leakage was monitored using a Model 3200 conductance instrument (YSI) every 5 h from 15 to 45 or 20 to 50 hpi. Leaf discs were kept at room temperature under constant 30 μmol s^−1^ m^−2^ light until electrolyte leakage measurements were conducted.

Statistical analyses were performed using the electrolyte leakage (μS/cm) data from the last timepoint collected. Normality of residuals was tested with the Shapiro-Wilk test and homogeneity of variances with the Levene’s test. If necessary, data were transformed using different power transformations to satisfy the ANOVA assumptions. Means were compared against each other using Tukey’s honestly significant difference test using α = 0.01 for all tests. All electrolyte leakage experiments were repeated at least once, and the two experiments were analyzed both separately and in a combined ANOVA using experiments as blocks. All statistical analyses were performed using SAS version 9.4.

### Protein analyses.

Protein was extracted from 0.2 g of flash-frozen *Nicotiana benthamiana* leaf tissue collected 24 hpi by grinding under liquid nitrogen with a mortar and pestle and adding 400 μl of extraction buffer (50 mM Tris-HCl, pH 7.4, 150 mM NaCl, 10 mM EDTA, pH 8.0, 10 mM dithiothreitol, 0.2% Triton X-100, 0.5% [wt/vol] polyvinylpyrrolidone (PVP), 1:100 protease inhibitor cocktail [Sigma P9599], and 1 mM phenylmethylsulfonyl fluoride). The lysate was cleared by centrifuging at 12,000 × *g* for 10 min at 4°C. Supernatant was collected, was transferred to a new 1.5-ml tube, and was centrifuged again at 12,000 × *g* for 10 min at 4°C. The final supernatant was collected and denatured at 65°C for 10 min in 1× Laemmli (−bromophenol blue). The total protein was quantified using the Pierce 660-nm protein assay with the addition of the ionic detergent compatibility reagent (Thermo Fisher).

Twenty micrograms of protein for each sample were loaded and resolved on a 10% sodium dodecyl sulfate-polyacrylamide gel electrophoresis (SDS-PAGE) gel. Proteins were then transferred to a polyvinylidene diflouride membrane overnight at 30 V. Membranes were washed with Tris-buffered saline with Tween 20 (TBST) and were blocked with 5% blotting-grade blocker (BioRad) in TBST. A dilution of 1:5,000 anti-GFP-horseradish peroxidase (HRP) (Miltenyi Biotec) antibody was used to detect GFP and GFP fusion proteins. 1:1,000 αDsRed (Santa Cruz Biotechnology) and 1:25,000 anti-mouse IgG-HRP (Sigma-Aldrich) antibodies were used to detect AvrSr35-mRFP fusion protein, and 1:10,000 anti-HA-HRP (Roche) antibody was used to detect AvrSr35-HA fusion protein. Blots were imaged using SuperSignal west femto maximum sensitivity substrate (Thermo Fisher) and a ChemiDoc (BioRad).

For full-length, autoactive Sr35-GFP constructs, the encoded protein was immunoprecipitated to facilitate detection. Protein was extracted from 0.5 g of flash-frozen *Agrobacterium*-infiltrated *Nicotiana benthamiana* leaf tissue by grinding under liquid nitrogen with a mortar and pestle and adding 1 ml of extraction buffer (same as described previously). The lysate was cleared by centrifuging at 12,000 × *g* for 10 min at 4°C. Supernatant was collected, was transferred to a new 1.5-ml tube, and was centrifuged again at 12,000 × *g* for 10 min at 4°C. GFP-Trap_A beads (ChromoTec) were washed twice by adding 1 ml of extraction buffer (without PVP), then, pelleting by centrifugation at 1,000 × g for 2 min. Seven hundred microliters of the final protein supernatant were combined with 20 μl of the washed beads and were turned end-over-end at 4°C for 2 h. From the final supernatant, 37.5 μl was retained as loading control. Beads were collected by centrifuging at 1,000 × *g* for 2 min. Flow-through was discarded, and beads were washed three times with 1 ml of extraction buffer (without PVP). Proteins were eluted from beads by heating at 65°C for 10 min in 1× Laemmli buffer. Loading samples were also denatured in this way. Samples were separated by SDS-PAGE and were processed for GFP immunodetection as described above.

Ponceau S stain was used to visualize the Rubisco large subunit protein across protein samples. Blots were stained with a Ponceau S and acetic acid solution (0.1% [wt/vol] Ponceau S [MP Biomedicals] and 5% [vol/vol] acetic acid) for 5 min. Blots were then rinsed with distilled water until bands were clearly visible and background was appropriately reduced. The stained blots were imaged using a ChemiDoc (BioRad).

### Transient expression in barley.

*Agrobacterium*-mediated transient expression in barley leaves was performed as described previously (Lu et al. 2016). Manchuria barley (*Hordeum vulgare* L. cultivar Manchuria) plants were grown in a growth chamber for 2 weeks at 23°C with 10 h of 100 μmol s^−1^ m^−2^ light. *Nicotiana benthamiana* (control) plants were grown for 3 weeks in growth chambers at 25°C with 85% humidity and 16 h of 100 μmol s^−1^ m^−2^ light. *Agrobacterium tumefaciens* AGL1 was transformed with *Sr35* and *AvrSr35* constructs. Fresh *Agrobacterium* cultures were grown overnight at 28°C in LB broth containing, per milliliter, 50 μg of kanamycin, 100 μg of ampicillin, and 100 μg of rifampicin. Bacterial cultures were pelleted by centrifugation, were resuspended in infiltration buffer (10 mM MES, pH 5.6, 10 mM MgCl_2_, and 400 μmol acetosyringone), and were adjusted to a final OD_600_ of 2.0. For Sr35-AvrSr35 coinfiltration experiments, each culture was adjusted to OD_600_ of 2.0 and was mixed in a 1:1 ratio. *Agrobacterium* were syringe-infiltrated into the second leaf of 12-day-old barley plants and into leaves of *N. benthamiana* as a positive control. After *Agrobacterium* infiltration, barley and *N. benthamiana* plants were placed at room temperature under constant 30 μmol s^−1^ m^−2^ light. *N. benthamiana* leaves were harvested and imaged at 48 or 72 hpi for macroscopic cell-death observations. For each construct, 20 leaves from 20 individual barley plants were harvested and were imaged 7 days postinfiltration for macroscopic cell-death observation.

## Supplementary Material

supplementary tables

supplementary figures
