# Supplementary material for: Dissection of Cell Death Induction by Wheat Stem Rust Resistance Protein Sr35 and Its Matching Effector AvrSr35
Source: Mol Plant Microbe Interact. Author manuscript; Available in PMC 2020 Jun 23. (PMC7309591; doi:10.1094/MPMI-08-19-0216-R)
Supplement: supplementary tables [file NIHMS1589401-supplement-supplementary_tables.pdf]

Supplementary Table S1. Primers used in this study.

Primer Name	Primer sequence 5' to 3'
Sr35gateway_F	GGGGACAAGTTTGTACAAAAAAGCAGGCTTCATGGAGATTGCCATGGGG
Sr35gateway_R	GGGGACCACTTTGTACAAGAAAGCTGGGTCCCATATATCGAGGATGGG
Sr35CCgateway_R	GGGGACCACTTTGTACAAGAAAGCTGGGTCGTCAACACTAGTATTAGC
Sr35NBgateway_F	GGGGACAAGTTTGTACAAAAAAGCAGGCTTCATGCCTCGCATGATGGCCTTG
Sr35NBgateway_R	GGGGACCACTTTGTACAAGAAAGCTGGGTCGGAAAGGGGTTCCATCCT
Sr35ARCgateway_R	GGGGACCACTTTGTACAAGAAAGCTGGGTCGGTATCCACATCTATCTT
Sr35LRRgateway_F	GGGGACAAGTTTGTACAAAAAAGCAGGCTTCATGACTAGGATGGAGCATATG
Sr35C319A_F	GTTAGTGTCTCTGAAGCAGCTTGCTCTTCTGAGGATG
Sr35C319A_R	CATCCTCAGAAGAGCAAGCTGCTTCAGAGACACTAAC
Sr35C649A_F	CAACTAAGACGTCTAATGGCTCTGTATGTTGATTATG
Sr35C649A_R	CATAATCAACATACAGAGCCATTAGACGTCTTAGTTG
Sr35D503V_F	GCTTGCCGTGTACATGTTATGGTGCTTGACCTC
Sr35D503V_R	GAGGTCAAGCACCATAACATGTACACGGCAAGC
Sr35K206R_F	GTGGGTTAGGCAGGACGACTCTTGC
Sr35K206R_R	GCAAGAGTCGTCCTGCCTAACCCAC
Sr35_TGA_gateway_R	GGGGACCACTTTGTACAAGAAAGCTGGGTCTCACCATATATCGAGGAT
Sr35CC_TGA_gateway_R	GGGGACCACTTTGTACAAGAAAGCTGGGTCTCAGTCAACACTAGTATTAGC
AvrSr35gateway_F	GGGGACAAGTTTGTACAAAAAGCAGGCTTCATGGCCATGAGGAACTTTGCTGC
AvrSr35gateway_R	GGGGACCACTTTGTACAAGAAAGCTGGGTCCAATTTGCCTTCATGAACATT

Supplementary Table S2. Statistical analysis of electrolyte leakage at 45 h by individual experiments and in a combined ANOVA using experiments as blocks. We compared all means against each other using Tukey's tests. Different letters indicate significant differences at $P < 0.01$. Results from Experiment 1 are shown in Figure 1C.

Construct	Exp. 1	$P < 0.01$	Exp. 2	$P < 0.01$	Combined	$P < 0.01$
1. Sr35:GFP D503V	66.3 ± 3.4	A	74.2 ± 3.8	A	70.2 ± 2.8	A
2. Sr35:GFP	23.5 ± 3.2	B	23.4 ± 1.8	B	23.4 ± 1.7	B
3. GFP:Sr35 D503V	8.7 ± 1.8	C	12.4 ± 1.8	BC	10.5 ± 1.4	C
4. GFP:Sr35	10.6 ± 1.2	C	11.3 ± 1.8	C	10.9 ± 1.0	C
5. GFP	7.4 ± 0.5	C	8.3 ± 0.7	C	7.9 ± 0.4	C
6. Empty vector	6.4 ± 0.6	C	9.1 ± 0.9	C	7.8 ± 0.7	C
Overall ANOVA [a]	$P < 0.0001$		$P < 0.0001$		$P < 0.0001$	

[a] A power transformation was used in the combined analysis to restore normality of residuals (Shapiro-Wilk test) and homogeneity of variances (Levene's test). Tukey P values are from the transformed data, but the means in the table are from untransformed data followed by the standard error of the means (s.e.m.). Individual experiments did not require transformation.

Supplementary Table S3. Sr35 truncations. Statistical analysis of electrolyte leakage at 50 h by individual experiments and in a combined ANOVA using experiments as blocks. We compared all the means against each other using Tukey's tests. Different letters indicate significant differences at $P < 0.01$. Results from Experiment 1 are shown in Figure 2C.

Construct	Exp. 1	$P < 0.01$	Exp. 2	$P < 0.01$	Combined	$P < 0.01$
1. WT	31.5 ± 3.1	A	29.8 ± 2.6	A	30.7 ± 1.9	A
2. CC	12.4 ± 1.0	B	10.4 ± 0.7	B	11.4 ± 0.7	B
3. LRR	12.5 ± 1.6	B	8.8 ± 0.7	BC	10.6 ± 1.1	B
4. NB	11.8 ± 1.8	B	8.4 ± 0.9	BC	10.1 ± 1.1	B
5. NB-ARC	12.9 ± 3.4	B	6.9 ± 0.1	BC	9.9 ± 1.9	B
6. CC-NB	10.1 ± 1.1	B	6.4 ± 0.8	BC	8.3 ± 1.0	B
7. NB-ARC-LRR	7.6 ± 1.8	B	8.5 ± 0.8	BC	8.0 ± 0.9	B
8. CC-NB-ARC	7.6 ± 1.1	B	5.5 ± 0.8	C	6.6 ± 0.7	B
9. Empty vector	10.4 ± 0.8	B	4.9 ± 0.7	C	7.7 ± 1.2	B
Overall ANOVA [a]	$P < 0.0001$		$P < 0.0001$		$P < 0.0001$	

[a] A square root transformation was used to restore normality of residuals (Shapiro-Wilk test) and homogeneity of variances (Levene's test) in the combined analysis and in Experiment 2. Tukey *P* values are from the transformed data, but all the means in the table are from untransformed data followed by s.e.m. Experiment 1 did not require transformation.

Supplementary Table S4. Sr35 auto-active mutant truncations. Statistical analysis of electrolyte leakage at 50 h by individual experiments and in a combined ANOVA using experiments as blocks. We compared all the means against each other using Tukey's tests. Different letters indicate significant differences at $P < 0.01$. Results from Experiment 1 are shown in Figure 3C.

Construct	Exp. 1	$P < 0.01$	Exp. 2	$P < 0.01$	Combined	$P < 0.01$
1. Complete D503V	77.3 ± 2.7	A	77.5 ± 5.6	A	77.4 ± 2.9	A
2. CC-NB-ARC D503V	53.7 ± 2.0	AB	56.5 ± 6.7	AB	55.1 ± 3.3	A
3. WT	23.1 ± 3.6	B	35.1 ± 4.5	B	29.1 ± 3.5	B
4. NB-ARC-LRR	9.7 ± 1.4	C	11.6 ± 1.7	C	10.7 ± 1.1	C
5. Empty vector	8.5 ± 1.1	C	7.3 ± 0.8	C	7.9 ± 0.7	CD
6. NB-ARC-LRR D503V	7.0 ± 0.5	C	8.5 ± 0.8	C	7.8 ± 0.5	CD
7. CC-NB-ARC	7.3 ± 1.2	C	8.3 ± 0.4	C	7.8 ± 0.6	CD
8. NB-ARC	7.2 ± 0.6	C	7.0 ± 0.3	C	7.1 ± 0.3	D
9. NB-ARC D503V	6.3 ± 0.3	C	6.6 ± 0.1	C	6.5 ± 0.2	D
Overall ANOVA [a]	$P < 0.0001$		$P < 0.0001$		$P < 0.0001$	

[a] A power transformation was used to restore normality of residuals (Shapiro-Wilk test) and homogeneity of variances (Levene's test) for the ANOVA. Tukey P values are from the transformed data, but all the means in the table are from untransformed data followed by s.e.m.

Supplementary Table S5. Sr35 wild type and auto-active mutants with and without mutations C319A and C649A in putative palmitoylation sites. Statistical analysis of electrolyte leakage at 45 h by individual experiments and in a combined ANOVA using experiments as blocks. We compared all the means against each other using Tukey's tests. Different letters indicate significant differences at $P < 0.01$. Results from Experiment 1 are shown in Supplementary Figure S3C.

Construct	Exp. 1	$P < 0.01$	Exp. 2	$P < 0.01$	Combined	$P < 0.01$
1. Sr35:GFP D503V	66.3 ± 3.4	A	77.6 ± 4.7	A	71.9 ± 3.4	A
2. $Sr35_{C319A/C649A}$:GFP D503V	61.2 ± 5.0	A	72.7 ± 3.7	A	66.9 ± 3.6	A
3. Sr35:GFP	23.5 ± 3.2	B	24.7 ± 4.3	B	24.1 ± 2.5	B
4. $Sr35_{C319A/C649A}$:GFP	20.9 ± 1.6	B	17.7 ± 2.7	B	19.3 ± 1.5	B
5. $Sr35_{K206R}$:GFP	8.7 ± 1.8	C	5.8 ± 0.7	C	7.3 ± 1.1	C
6. Empty vector	6.4 ± 0.6	C	5.0 ± 0.4	C	5.7 ± 0.4	C
ANOVA [a]	$P < 0.0001$		$P < 0.0001$		$P < 0.0001$	

[a] A square root transformation was used to restore normality of residuals (Shapiro-Wilk test) and homogeneity of variances (Levene's test) in all ANOVAs. Tukey P values are from the transformed data, but all the means in the table are from untransformed data followed by s.e.m.

Supplementary Table S6. Sr35 and AvrSr35 co-infiltrations. Statistical analysis of electrolyte leakage at 50 h by individual experiments and in a combined ANOVA using experiments as blocks. We compared all the means against each other using Tukey's tests. Different letters indicate significant differences at $P < 0.01$. Avr= AvrSr35-SP+mRFP (effector without the signal peptide and with a Cterminal monomeric Red Fluorescent Protein tag). Results from Experiment 2 are shown in Figure 5C.

Construct	Exp. 1	$P < 0.01$	Exp. 2	$P < 0.01$	Combined	$P < 0.01$
1. Avr + Sr35:GFP	65.7 ± 1.3	A	85.5 ± 3.2	A	75.6 ± 4.1	A
2. Avr + Sr35:GFP D503V	69.1 ± 7.4	A	81.9 ± 3.2	A	75.5 ± 4.5	A
3. Sr35:GFP D503V	68.6 ± 1.0	A	79.4 ± 6.4	A	74.0 ± 3.6	A
4. Sr35:GFP	23.1± 1.7	B	27.7 ± 1.2	B	25.4 ± 1.3	B
5. Avr + Sr35CCNBARC	10.9 ± 0.6	C	8.1 ± 0.5	C	9.5 ± 0.7	C
7. Avr + Avr[a]	9.3 ± 0.6	CD	6.4 ± 0.8	C	7.9 ± 0.7	C
6. Avr + GFP:Sr35	8.4 ± 1.5	CD	8.7 ± 1.3	C	8.5 ± 0.9	C
8. Sr35 CCNBARC	6.8 ± 0.7	CD	8.7 ± 0.9	C	7.7 ± 0.6	C
9. Avr	6.9 ± 0.5	CD	6.6 ± 0.8	C	6.8 ± 0.4	C
10. GFP:Sr35	6.4 ± 0.6	D	6.4 ± 0.5	C	6.4 ± 0.4	C
Overall ANOVA [b]	$P <$ 0.0001		$P <$ 0.0001		$P <$ 0.0001	

[a] Avr+Avr co-infiltration control corresponds to AvrSr35 infiltrated at double the optical density of Avr.

[b] A log+1 transformation was used to restore normality of residuals (Shapiro-Wilk test) and homogeneity of variances (Levene's test) in all the ANOVAs. Tukey P values are from the transformed data, but all the means in the table are from untransformed data followed by s.e.m.
