# Supplementary material for: Dissection of Cell Death Induction by Wheat Stem Rust Resistance Protein Sr35 and Its Matching Effector AvrSr35
Source: Mol Plant Microbe Interact. Author manuscript; Available in PMC 2020 Jun 23. (PMC7309591; doi:10.1094/MPMI-08-19-0216-R)
Supplement: supplementary figures [file NIHMS1589401-supplement-supplementary_figures.pdf]

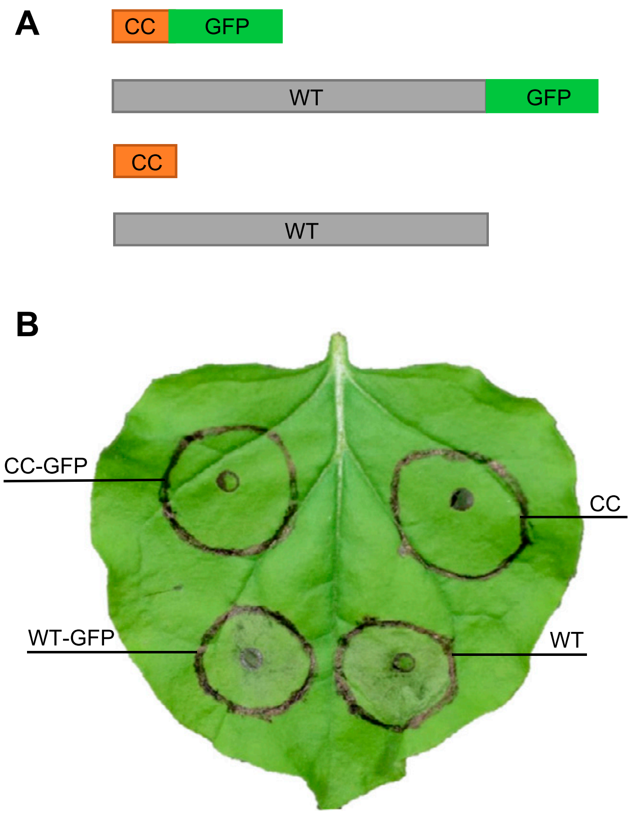

Supplementary Figure S1. The addition of a C-terminal GFP tag did not alter the induction of cell death by the Sr35 CC domain and the Sr35 complete protein in *N. benthamiana.* **A,** Models of the Sr35 CC domain and the complete Sr35 protein with and without a GFP tag. The CC domain corresponds to the first 155 amino acids of Sr35 protein. **B,** Macroscopic cell death observed in *N. benthamiana* leaves 48 hpi with *Agrobacterium.* CC = coiled-coil, WT= wild-type, GFP= green fluorescent protein.

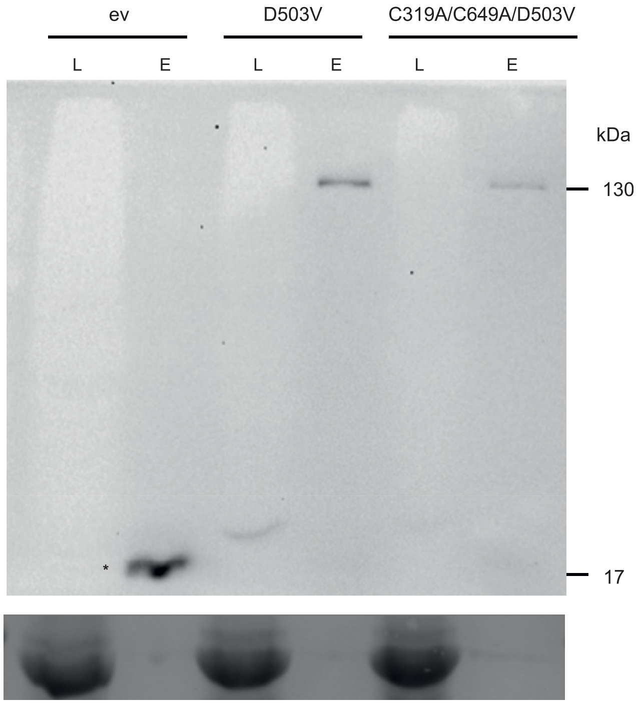

Supplementary Figure S2. Auto-active (D503V) full-length Sr35 proteins were expressed at the expected size. Protein was extracted from *N. benthamiana* leaves 24 hpi. Sr35 D503V auto-active and C319A/C649A/D503V constructs with C-terminal GFP tags were immunoprecipitated with GFP-Trap beads. The blot was stained with Ponceau S (below) to reveal bands corresponding to Rubisco's large subunit protein as loading control. ev = empty vector; L = loading control sample; E = GFP immunoprecipitation elution. A cross-reacting band* was observed around 17kDa in the ev elution.

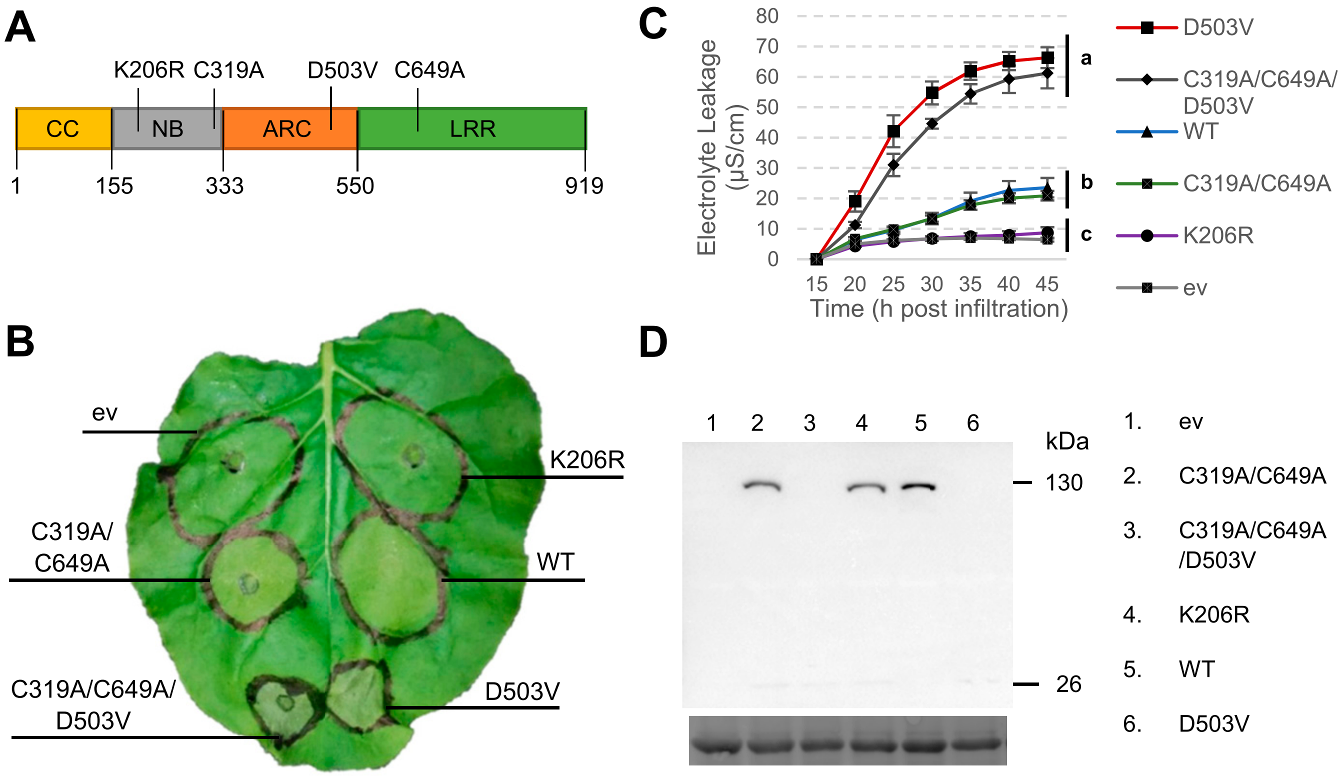

Supplementary Figure S3. Mutation of predicted palmitoylation sites in Sr35 did not affect the induction of cell death. **A,** Model of Sr35 protein with the amino acid numbers used to delineate the domains and/or motifs listed below. CC = coiled-coil, NB = nucleotide binding, NB-ARC = nucleotide binding adaptor, and LRR = leucine-rich repeat. Positions of auto-active mutation D503V, P-loop mutation K206R, and predicted palmitoylation site mutations C319A and C649A are marked above the model. **B,** Macroscopic cell death observed when Sr35 WT and its mutants were transiently expressed in *Nicotiana benthamiana.* Leaves were imaged 48 hpi with *Agrobacterium*. All proteins were C-terminally tagged with GFP. ev = empty vector construct. **C,** Electrolyte leakage for all constructs was monitored from 15 to 45 hpi. Error bars represent standard error based on four biological replicates per construct. Different letters indicate significantly different groups of means based on Tukey's HSD test ($\alpha = 0.01$) performed at the last time point (statistical analyses in Supplementary Table S5). **D,** GFP-HRP Western blot showing that all constructs expressed proteins of the expected sizes. 20µg of protein extracted from *N. benthamiana* tissue at 24 hpi was analyzed for each sample. No protein was observed for both Sr35 D503V-GFP and Sr35 C319A/C649A/D503V, presumably due to strong cell death induced by these constructs at this time point. However, these proteins were detected after immunoprecipitation (Supplementary Fig. S2). The blot was also stained with Ponceau S (below) to reveal bands corresponding to Rubisco's large subunit protein as loading control.
